# *ECLed*– a tool supporting the effective use of the SNOMED CT Expression Constraint Language

**DOI:** 10.1186/s13326-025-00344-3

**Published:** 2026-01-06

**Authors:** Tessa Ohlsen, André Sander, Josef Ingenerf

**Affiliations:** 1 Institute of Medical Biometry and Statistics, Section for Clinical Research IT, University of Luebeck, University Hospital Schleswig-Holstein, Ratzeburger Allee 160, 23562 Luebeck, Germany; 2 Institute of Medical Informatics, University of Luebeck, Luebeck, Germany; 3 ID Berlin GmbH & Co KGaA, Berlin, Germany

**Keywords:** SNOMED CT, Expression Constraint Language, HL7 FHIR, Semantic Interoperability, Terminology

## Abstract

**Background:**

The Expression Constraint Language (ECL) is a powerful query language for SNOMED CT, enabling precise semantic queries across clinical concepts. However, its complex syntax and reliance on the SNOMED CT Concept Model make it difficult for non-experts to use, limiting its broader adoption in clinical research and healthcare analytics.

**Objective:**

This work presents *ECLed*, a web-based tool designed to simplify access to ECL queries by abstracting the complexity of ECL syntax and the SNOMED CT Concept Model. *ECLed* is aimed at non-technical users, enabling the creation and modification of ECL queries and facilitating the querying of patient data coded with SNOMED CT.

**Methods:**

*ECLed* was developed following a detailed requirements analysis, addressing both functional and non-functional needs. The tool supports the creation and editing of SNOMED CT ECL queries, integrates a processed Concept Model, and uses FHIR terminology services for semantic validation. Its modular architecture, with a frontend based on Angular and a backend on Spring Boot, ensures seamless communication through RESTful interfaces.

**Result:**

*ECLed* demonstrated high usability in a user survey. Technical validation confirmed that it reliably generates and edits complex ECL queries. The tool was successfully integrated into the *DaWiMed* research platform, enhancing clinical analysis workflows. It also worked effectively with clinical data in FHIR format, although scalability with larger datasets remains to be tested.

**Discussion:**

*ECLed* overcomes the limitations of existing ECL tools by abstracting the complexity of both the syntax and the SNOMED CT Concept Model. It provides a user-friendly solution that enables both technical and non-technical users to easily create and edit ECL queries.

**Conclusion:**

*ECLed* offers a practical, user-friendly solution for creating SNOMED CT ECL queries, effectively hiding the underlying complexity while optimizing clinical research and data analysis workflows. It holds significant potential for further development and integration into additional research platforms.

**Supplementary Information:**

The online version contains supplementary material available at 10.1186/s13326-025-00344-3.

## Introduction

### Background

The structured and cross-institutional use of clinical data is becoming increasingly critical in healthcare and medical research. In today’s digital healthcare environment, the ability to accurately capture, integrate, and analyse medical information is fundamental to data-driven decision-making. Whether in direct patient care, clinical research, or quality assurance, data-driven insights are key to improving patient outcomes, streamlining operations, and fostering medical innovation. However, for clinical data to be truly useful, it must be semantically interpretable, enabling automatic processing, analysis, and secure exchange [1–3]. This is where powerful coding systems like SNOMED Clinical Terms (SNOMED CT) come into play. SNOMED CT is one of the most comprehensive and internationally recognized clinical terminologies. It enables the standardized representation of clinical concepts across various healthcare systems and countries. In addition to functioning as a terminology, SNOMED CT acts as a formal ontology, providing a logically structured, computable representation of medical knowledge. Together, these features establish a high level of semantic standardization which ensures that medical concepts can be uniformly interpreted by machines, supporting clinical decision-making, decision support systems, and supports interoperable data exchange [3]. To fully unlock its potential, SNOMED CT provides the Expression Constraint Language (ECL), a machine-processable language designed for querying SNOMED CT’s concepts based on a grammar. ECL allows users to define precise queries based on hierarchical relationships, attributes, and logical combinations, making it ideal for integration into tools and software systems [4]. ECL can be applied in three key areas:

1. **Basic use case: interactive SNOMED CT content queries**
  The most common use case for ECL is the interactive exploration of SNOMED CT content [4–6]. This is typically done through web-based tools like the *SNOMED CT Browser* [5, 7] or *WASP* [8]. These tools allow users to retrieve relevant clinical concepts through ECL, based on semantic attributes, hierarchical relationships, or their combinations. This functionality is particularly helpful for tasks such as terminology familiarization, identifying suitable codes for documentation [3]. A typical ECL query, for instance, might retrieve all disorders associated with infarct morphology or caused by myocardial infarction [4]:

* << 64572001 |Disease (disorder)|*:

*{ 116676008 |Associated morphology (attribute)| =*

*<< 55641003 |Infarct (morphologic abnormality)| OR*

*42752001* *|Due to (attribute)| =*

*<< 22298006 |Myocardial infarction (disorder)|}.*

In the SNOMED CT International Edition, 2025-01-01, this query returns 283 concepts, including *Cerebral infarction*, *Myocardial infarction*, and *Ventricular aneurysm due to and following acute myocardial infarction*. While these content-based queries are semantically rich and widely used, they remain largely disconnected from real patient data.

2. **Technical use cases: terminology engineering and interoperability**
  Beyond interactive use, ECL plays a crucial role in defining intentional ReferenceSets, specifying Content Models, and linking terminologies to external data models like HL7 FHIR [4]. For example, when defining a FHIR ValueSet, ECL ensures that all relevant descendant concepts are included dynamically, ensuring consistency and semantic accuracy. These technical use cases help bridge the gap between clinical terminology and its application in decision support, clinical pathways, and quality metrics [3].

3. **Semantic use cases: querying SNOMED CT–coded patient data**
  The most impactful, yet currently least widespread, use of ECL is querying patient data encoded with SNOMED CT. This includes applications like structured data entry, NLP-based coding, terminology mapping, and clinical data analytics. When ECL is applied directly to patient-level data, such as SNOMED CT concepts in electronic health records, it enables precise cohort selection, clinical feature analysis, and rule-based decision support [3]. A concrete example of this is the *DaWiMed* research platform developed by ID GmbH & Co. KGaA [9], which combines structured clinical documentation with analytical tools and supports standards like ICD-10, OPS, and SNOMED CT. However, the availability of SNOMED CT–coded patient data remains limited in many healthcare systems, primarily due to reliance on legacy coding schemes. This limitation restricts the broader application of ECL in clinical decision-making and research.

Despite its expressive power, ECL can be challenging for many users. It is based on a formally defined grammar and requires a solid understanding of the SNOMED CT Concept Model [3]. Without prior experience in clinical terminologies, creating syntactically correct and semantically valid queries manually can be difficult. To lower these barriers, the web application *ECLed* (pronunciation “ee-see-led“) was developed. Its goal is to support the efficient and user-friendly creation and maintenance of ECL queries based on version 2.2. The core component is a user interface that allows users to build complex queries without directly writing ECL syntax. While not all components of the full ECL specification have been implemented, the development focuses on key features identified through a requirements analysis.

*ECLed* targets both terminology experts who want to create precise queries and users with less technical expertise, such as those working in research, quality assurance, or clinical decision support. Moreover, *ECLed* is integrated into the *DaWiMed* research platform, enabling seamless query creation within this environment, enhancing the platform’s utility for clinical data analysis and research.

### Related work

**Fig. 1:**
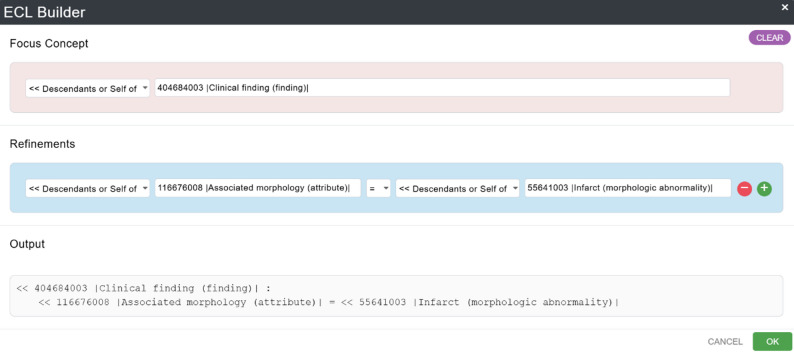
Extract from the *ECL Builder* included in the SNOMED CT Browser [7]

**Fig. 2:**
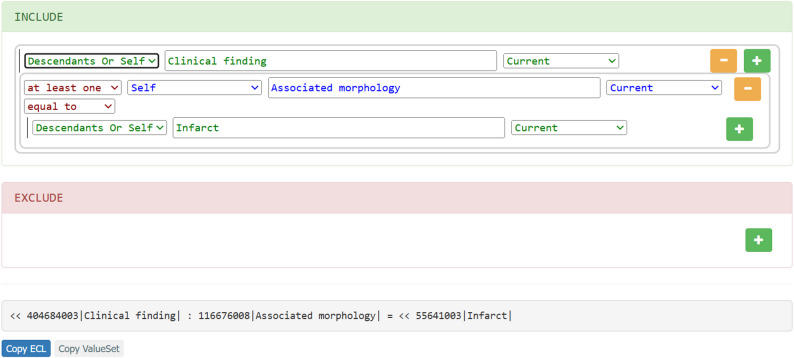
Extract from the *Shrimp* tool

While existing work such as Giménez-Solano et al. [10] explores the use of ECL, our approach focuses on the practical support of non-technical users in formulating semantic queries within clinical data contexts. *ECLed* addresses a different challenge, namely the barrier posed by complex terminology languages such as ECL in medical research and complements existing work by providing a user-centred solution for semantic data analysis. Currently, only a few tools support the use of SNOMED CT via the Expression Constraint Language. Notable examples include the a *ECL builder* included in the *SNOMED CT Browser* [7], provided by SNOMED International, and *Shrimp* [11], developed by the Commonwealth Scientific and Industrial Research Organisation (CSIRO). *ECL Builder* [7] (see Fig. 1) follows a strictly SNOMED CT Concept Model–conformant approach. Users define one or more focus concepts and can then only apply attributes that are permitted within the corresponding domain. However, these attributes are not presented in a drop-down menu. Instead, users must know the exact attribute names (e.g., *Associated morphology*) and, more importantly, understand the underlying Concept Model to use the tool effectively. Another limitation is that the results of a query are not displayed in real time; they only become visible once the query has been fully constructed and explicitly executed. The expressive power of *ECL Builder* is also rather restricted. For instance, only single concepts can be specified as attribute values. Combining multiple values using logical operators (*AND*, *OR*, *MINUS*) or structuring more complex expressions with parentheses is not supported. Nested expressions are likewise not possible. Furthermore, syntax validation is rudimentary, which can lead to erroneous placeholder expressions such as *“<< 71388002 |Procedure (procedure)|”* when no matching concept is found. *Shrimp* [11] (see Fig. 2), in contrast, emphasizes interactivity and expressive flexibility. As soon as a new element is added to a query, matching concepts are displayed immediately. After selecting a focus concept, users can also construct more complex queries, which makes the functionality richer but also more demanding in terms of usability. A key distinction from the *ECL Builder* is that *Shrimp* does not enforce Concept Model conformance. For example, when choosing *Clinical finding* as a focus concept, it is still possible to assign an attribute such as *Procedure site*, even though this is not valid for that domain. Likewise, the attribute value selected may be incompatible. The only feedback on such inconsistencies is the absence of matching results, which means that knowledge of the Concept Model remains essential for correct usage. Beyond this, Shrimp provides additional features such as working with *Historical Association Reference Sets* and *History Profiles*, defining exclusions (to represent negations), and specifying cardinalities for relationships. In summary, the *ECL Builder* ensures robustness by adhering strictly to the Concept Model but is limited in expressive power. *Shrimp*, on the other hand, offers an interactive interface and a broad range of functionalities for constructing complex queries, though it requires a high level of conceptual knowledge and entails the risk of producing invalid queries.

In previous work, the authors developed the web application *WASP* [8] for creating postcoordinated SNOMED CT expressions (PCE). This approach shares methodological similarities with the Expression Constraint Language, particularly in terms of grammar and the Concept Model. However, ECL is more complex, presenting new challenges that were not addressed by creating PCEs, such as handling parenthesized expression constraints with multiple logical operations and the representation of complex *Reverse* functions. Insights from a usability study conducted for *WASP* informed the development of *ECLed*: interface elements that received positive feedback were retained; while weaknesses, such as difficulties in finding appropriate concepts, were addressed to improve the user experience.In summary, the current project builds on these experiences and focuses on the more complex requirements of ECL. It also considers improvements to existing tools such as the *SNOMED CT Browser* and *Shrimp*, as well as enhancements to *WASP*, particularly in terms of usability and architecture.

The current project builds on these experiences and addresses the more complex requirements of the Expression Constraint Language (ECL), while also considering improvements to existing tools such as the SNOMED CT Browser, Shrimp, and the architectural aspects of WASP. Similar challenges are well known from relational databases and SQL, where users must master a complex syntax and understand the underlying schema. To mitigate this, tools such as visual query builders and syntax checkers have been developed to support the construction of valid queries [12]. The situation is analogous in the grammar of ECL, where the Concept Model defines valid combinations of concepts and attributes. Our tool follows a similar rationale: it lowers the entry barrier, prevents invalid queries, and guides users in working effectively with a complex data model.

## Implementation

### Requirements analysis

**Fig. 3:**
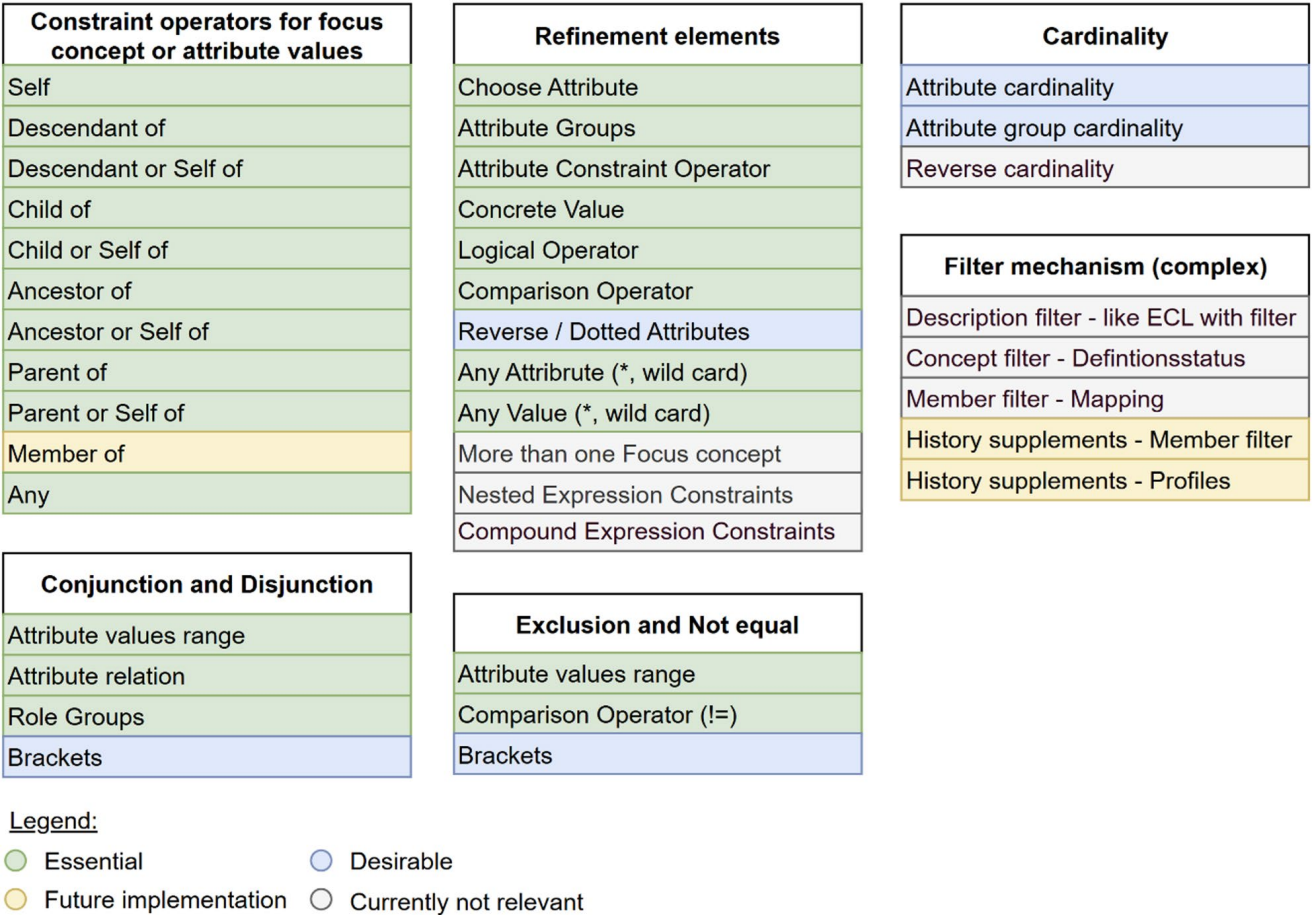
Categorization of ECL grammar components based on expert prioritization according to the ECL Guide [4]

In the development of the tool *ECLed*, it was essential to clearly define both the functional and non-functional requirements. These requirements form the foundation for designing a tool that meets the needs of its target users while ensuring high quality and usability. International standards, such as ISO/IEC 25010 [13], were referenced to ensure that the tool fulfils the necessary quality characteristics. The non-functional requirements focus on software quality aspects such as usability, performance, and maintainability, aiming to deliver a reliable and sustainable solution. They ensure that *ECLed* is not only functionally capable but also meets the practical demands placed on modern software solutions. In contrast, the functional requirements define the specific features and capabilities the tool must offer to support users in the creation and maintenance of SNOMED CT ECL queries. These requirements concentrate on the functionalities necessary for efficient and error-free work with the tool. The most important non-functional and functional requirements for *ECLed* are summarized in Table 1. In addition, the SNOMED ECL grammar was analyzed in collaboration with domain experts with in-depth knowledge of SNOMED CT, using the ECL Guide [4] as a reference. The objective of this analysis was to identify and understand the structural components of the ECL syntax. Following this, each component was examined in detail and prioritized based on its relevance and necessity for the tool’s initial and future development. The results of this prioritization are presented in Fig. 3, where the keywords reflect the section headings of the ECL Guide [4]. The components were classified into four categories: essential, desirable, identified for future implementation, and currently not relevant.

**Table 1:** Functional and non-functional requirements

	ID	Feature	Description
**Non-functional**	N1	Usability	*ECLed* must offer an intuitive and user-friendly interface that allows users without deep technical knowledge to easily create SNOMED CT ECL queries.
	N2	Functionality	*ECLed* must reliably and accurately provide all required functionalities, such as visual query building, syntax validation, and semantic checking in accordance with the SNOMED CT specification.
	N3	Performance	The tool must deliver fast response times during real-time validation and querying of FHIR servers, even for more complex queries.
	N4	Maintainability	The architecture of the tool must be designed in such a way that future extensions or features can be integrated with minimal effort.
	N5	Portability	*ECLed* must be cross-platform compatible with common web browsers and functional on various end devices.
**Functional**	F1	Creation of ECL queries	*ECLed* enables users to create ECL queries without having to manually enter the ECL syntax. It should also allow users to choose between German and English language settings.
	F2	Semantic validation	*ECLed* uses SNOMED CT’s Machine Readable Concept Model (MRCM) to ensure that created queries are semantically correct and use only valid concepts and relationships.
	F3	Integration of FHIR termino-logy services	*ECLed* can integrate different FHIR-based terminology server and the corresponding terminology services to retrieve and validate concepts and codes in real time. This ensures that users are working with current and valid terms.
	F4	Search of terms	*ECLed* provides a search function that allows users to find specific SNOMED CT concepts for use in their queries. Searches can be conducted using SNOMED CT Identifiers or concept names.
	F5	Import/export of ECL queries	*ECLed* supports the import and export of ECL queries to ensure reusability and integration with other systems.

### Expression Constraint Language

**Fig. 4:**
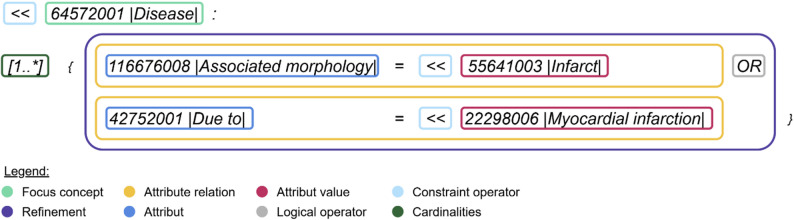
Example of an ECL query with visualized components

The SNOMED CT Expression Constraint Language [7] specification defines the syntax for defining expression constraints or query to precisely query concepts within the SNOMED CT terminology. It thus provides the foundation for structured and semantically accurate interaction with the terminology. An example query and its components are illustrated in Fig. 4.

The query is based on a focus concept (green), representing the simplest form of an ECL query. To capture more complex clinical scenarios, the focus concept can be refined (violet) by adding attribute relationships constraints (yellow). Each attribute relationship consists of an attribute (dark blue) and one or more attribute value constraints (red). Logical operators (grey) can be applied in different contexts:

- OR and AND can be applied between relationships,
- OR, AND, and MINUS can be used between attribute values constraints or multiple focus concepts.

Attribute relationship constraints can be grouped into role groups using curl. These are a syntactic construct that indicate that the contained attribute relationship constrains must be matched within the same role group.

Wildcards (*) can be used in place of concrete concepts, enabling more general and flexible query patterns. In addition, constraint operators (light blue) in ECL define the scope of concepts to be returned. Depending on the constraint operator, they can either broaden or narrow the result set. For example, the constraint *< < 55641003 |Infarct (morphologic abnormality)|* includes both the specified concept and all its descendants. Beyond defining the scope of concepts, ECL also allows the specification of cardinalities for attributes and relationships (dark green). These define the minimum and maximum number of times a particular attribute or relationship may occur within a concept definition. They can be applied either to a role group as a whole or to an individual attribute relationship, depending on the modelling need. By default, the cardinality is [1..*], meaning that at least one instance must be present, with no upper limit unless explicitly specified. In addition to the default cardinality [1..*], ECL also supports special cardinality constraints such as *[0..0]*. This indicates that a particular attribute or relationship must not occur at all. ECL also supports the use of filters, and the integration of history supplements.

The ECL is formally defined using Augmented Backus-Naur Form (ABNF). In this project, it serves as the foundation for the development of a user interface that translates user input into syntactically correct ECL queries and parses existing queries for further editing. This is supported using the open-source Java library *SNOMED CT Expression Constraint Language Parser* [14], provided by SNOMED International. This library enables validation, interpretation, and processing of ECL queries in accordance with the official specification and forms a central technical component of the ECL editor developed in this project. Additionally, the *ANother Tool for Language Recognition (ANTLR)* library [15] (version 4.5.3) is employed to facilitate the parsing and generation of abstract syntax trees, enhancing the overall performance and flexibility of the query processing framework.

### Processed Concept Model

**Fig. 5:**
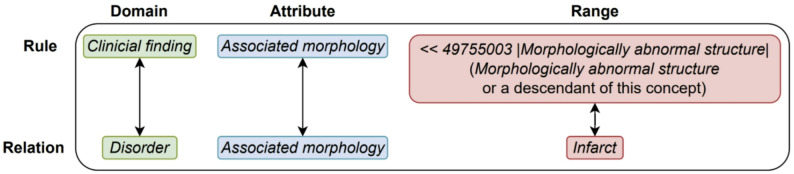
Domain and range example for the SNOMED CT attribute *Associated morphology*

The Concept Model describes the structure of SNOMED CT concepts and post-coordinated expressions through formal logical rules and specific guidelines ensuring their semantic correctness. These rules ensure a coherent and consistent representation of queries. For each of the over 120 attributes within SNOMED CT, a “Domain” and “Range” are defined (see Fig. 5):

- **Domain**: Refers to a set of SNOMED CT concepts whose meaning can be defined using a specific set of attributes. Domains are not limited to top-level hierarchies, such as *Clinical finding*, and may also be defined through reference sets, such as the *Lateralizable Body Structure ReferenceSet*.
- **Range**: Describes a subset of SNOMED CT concepts recognized as valid values for a specific attribute. For example, the attribute *Associated morphology* is valid only for specific morphological changes, such as *Infarct*.

Additionally, the Concept Model specifies the cardinality of attributes and determines whether attributes must be grouped [3, 16, 17].

**Fig. 6:**
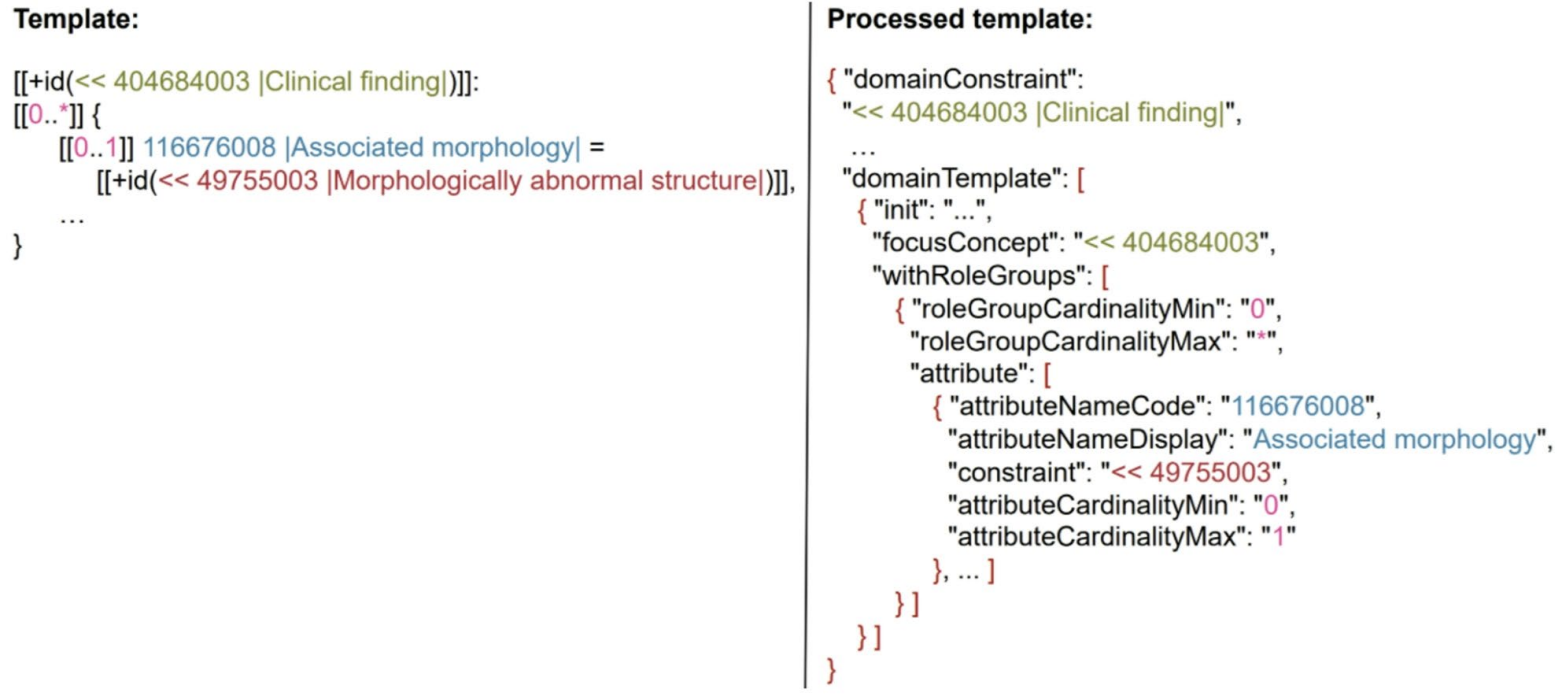
Extract from the processed MRCM in JSON format, which proves to be advantageous for further processing

For each SNOMED CT edition and version, a machine-readable Concept Model (MRCM) is provided in the RF2 files [18]. In this work, we use the MRCM Domain ReferenceSet of the International Edition, 2025-01-01. This ReferenceSet is provided as a text file, in which each of the 19 domains is described by a detailed entry. Each of these entries contains all relevant information, including a template, which is of particular importance for this work [18, 19]. An excerpt from such a template is shown on the left side of Fig. 6. The syntax of the templates is based on Expression Template Language. The template defines the relevant attributes, their cardinalities, and value ranges using ECL for each attribute, thereby ensuring the Concept Model-valid correctness of the expression constraints.

To enhance performance and efficiency, an algorithm was developed in earlier work [17] that decomposes each domain and its associated pre-coordinated template into individual components. This information is structured using JavaScript Object Notation (JSON), enabling efficient and systematic processing of template elements in subsequent steps. An excerpt of the JSON structure is shown on the right side of Fig. 6, and the complete document is available on Gitlab [20]. This processed MRCM forms the basis for generating semantically or Concept Model valid ECL queries. While this functionality is also supported by SNOMED International’s ECL builder, our model likewise enables applications to guide users during query creation by dynamically restricting selectable attributes and permissible values according to the selected focus concept. When a focus concept is chosen, the corresponding domain is automatically identified, and only the attributes defined for that domain and value ranges defined for that attribute are made available to ensure Concept Model consistency. This functionality is illustrated by the following example: if *Disease* is selected as the focus concept, the system determines the associated domain, *Clinical finding*, which includes 18 attributes, such as *Associated morphology*. If the user selects this attribute, the value range ensures that semantically inappropriate concepts like *Chromium-cobalt alloy* are excluded. In summary, the use of JSON allows for efficient, structured data representation and serves as a foundation for applications that support the construction of syntactically correct and semantically sound ECL query.

### FHIR terminology server and services

A central component of *ECLed* is the integration of a Health Level Seven (HL7) Fast Healthcare Interoperability Resources (FHIR) terminology server, which is accessed via the standardized FHIR terminology services defined by the HL7 FHIR specification [1]. These services enable effective, context-sensitive use of SNOMED CT and form the foundation for semantically precise and rule-compliant processing of terminology data within *ECLed*. The terminology services used in *ECLed* are summarized in Table 2. The communication with the terminology server primarily takes place via POST requests. To support the construction of request bodies and the structured processing of server responses, the open-source Java library *HAPI FHIR* (version 7.2.2.) [21] is used. This work utilizes two different terminology servers: *Ontoserver* [22] (CSIRO, version 6.14.3) and *SNOWSTORM* [23] (SNOMED International, version 10.7.0). Both servers offer the flexibility and performance required for dynamic querying, semantic validation, and structured analysis of concepts and their interrelations. To enable this, the deployed terminology server must have at least one SNOMED CT edition in a specific version available. In this work, the SNOMED CT International Edition, 2025-01-01, was used.

**Table 2:** FHIR terminology services used in *ECLed*

Service	Description	Use case in ECLed
*$lookup*	Retrieves properties of a concept based on a CodeSystem (e.g., display name, attribute relationships)	Display of concept names and identification of permissible attribute relationships
*$expand*	Resolves a ValueSet – defined via ECL or filters – into a concrete set of concepts	Determines allowed concepts for attribute values, e.g., using ECL queries with name-based filtering, or to retrieve the set of concepts resulting from a user-defined ECL query.
*$subsumes*	Checks hierarchical relationships (is-a) between two concept codes within a CodeSystem	Determines the valid Concept Model Domain for a given focus concept based on its position in the hierarchy

### FHIR Search

The main goal of *ECLed* is to support the creation of ECL queries for searching and defining a set of pre-coordinated concepts. Additionally, *ECLed* can also be used to find SNOMED CT-coded patient data. For this purpose, the *FHIR Search API* [24] is utilized. This data is hosted on a local *HAPI FHIR* server (Smile Digital Health, version 7.6.0) and consists of a test set of synthetic resources such as *Patient* and *Condition*. After creating an ECL query, a *$expand* operation is first performed using a FHIR terminology server to fully resolve all SNOMED CT concepts referenced by the query. The codes are combined using commas (*OR* operation), as per *FHIR Search* syntax, and sent via POST using the *_search* parameter to find matching cases. To optimize performance, a custom mapping between SNOMED CT Concept Model Domains and the corresponding FHIR resource types is used. This allows the search to be restricted to those resource types that are semantically relevant to the selected focus concept.

For instance, concepts from the domains *Disease (disorder)*,* Clinical finding (finding)*,* Finding with explicit context (situation)*, or *Situation with explicit context (situation)* are often mapped to the FHIR *Condition* resource. However, depending on the clinical context, such concepts may also be represented using other FHIR resources, such as *Observation*,* AllergyIntolerance*,* Procedure*, or *FamilyMemberHistory*. This feature is currently in an exploratory development stage and is being evaluated using a limited, synthetic test data environment.

### Tool architecture

**Fig. 7:**
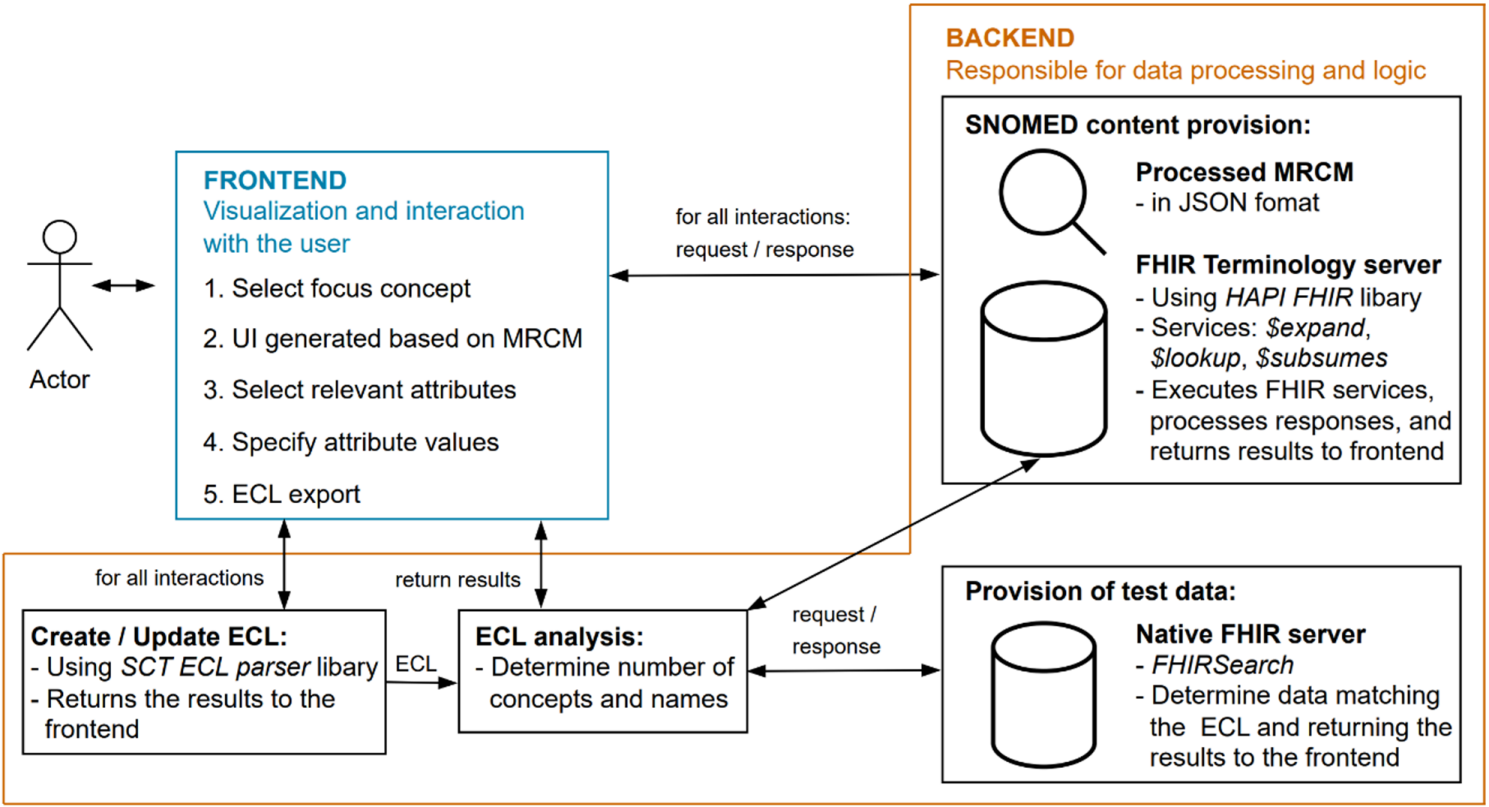
System architecture comprising an Angular frontend and a Spring Boot backend for ECL query construction

The system architecture (see Fig. 7) is based on a distinct separation between an Angular frontend (version 18) and a Java-based Spring Boot backend (version 3.3.2, Java 17). The goal of the application is to provide an intuitive interface for constructing SNOMED CT Expression Constraint Language queries. The frontend guides users through a step-by-step process in which a focus concept is selected, relevant attributes are defined, and corresponding values are specified. To support these semantic selection steps, the system utilizes FHIR terminology services – namely *$expand*, *$lookup*, and *$subsumes* – which are provided via an embedded terminology server based on the HAPI FHIR library. For this, the terminology server must have at least one SNOMED CT edition with a specific version available. The user interface is generated dynamically, based on Machine Readable Concept Model rules processed within the backend. The backend also includes modules for creating and analysing ECL queries, which determine the number of matching concepts and retrieve their human-readable labels. In addition, a native FHIR server is available to apply the generated ECL queries to real-world test data. Communication between frontend and backend is handled via RESTful interfaces within the Spring Boot application. The modular architecture allows for flexible extension and reusability of individual components across various use cases. The entire system is containerized using Docker, allowing for seamless deployment and management. Configuration parameters, such as the terminology server’s URL (e.g., https://r4.ontoserver.csiro.au/fhir) and the combined SNOMED CT version and edition (e.g., http://snomed.info/sct/900000000000207008/version/20250101*)*, are specified in the *config.env* file, ensuring flexibility and ease of setup across different environments.

## Results

### Web application *ECLed*

#### ECL query creation

**Fig. 8:**
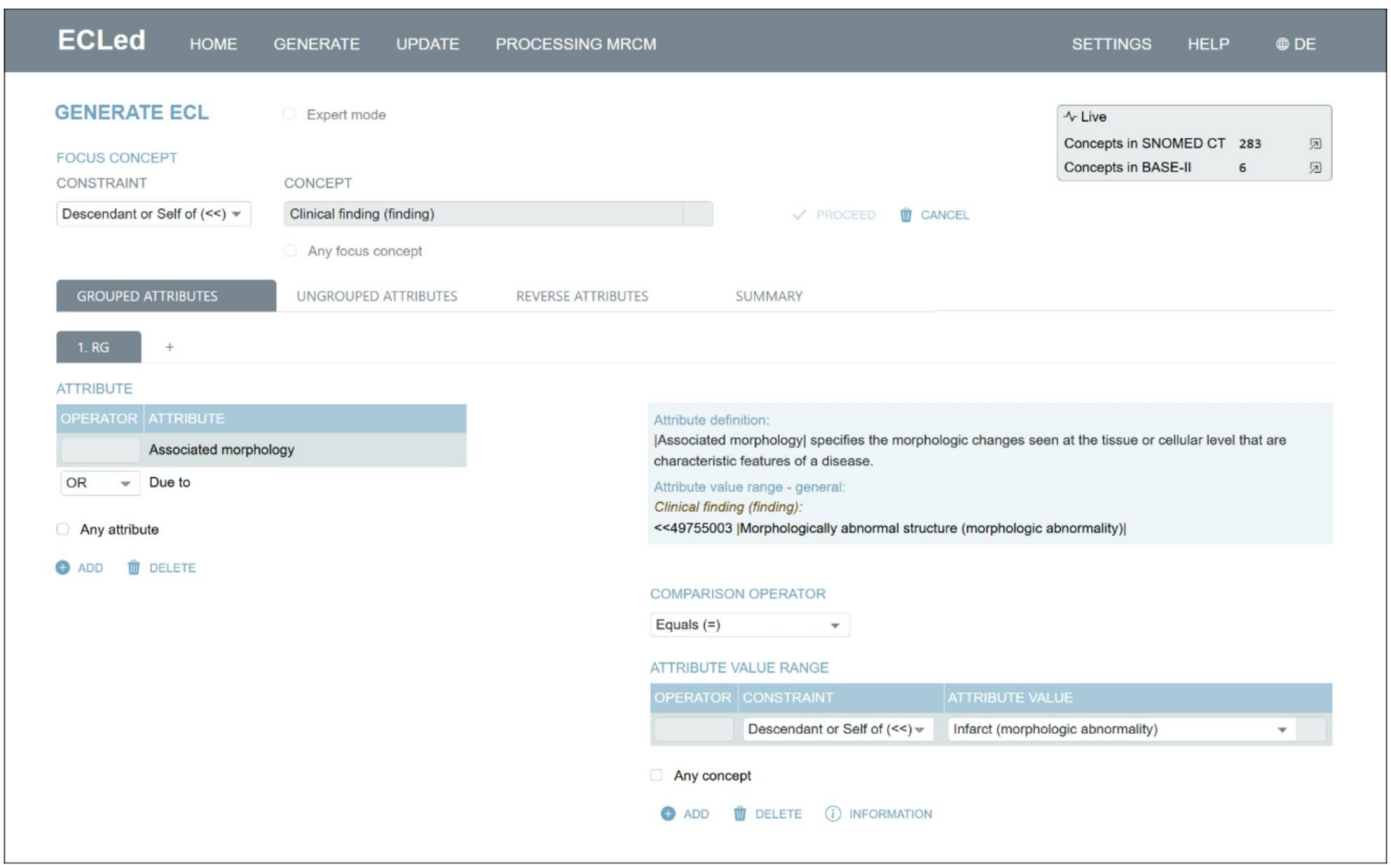
Extract from *ECLed* to generate an ECL query

The user selects an appropriate focus concept from a large set of SNOMED CT concepts, tailored to their specific use case. This concept serves as the semantic foundation for the ECL query to be created. An example of a focus concept is *Disease* (see Fig. 8). To initiate the selection, the user enters a search term, such as *“dis”*, into an input field. Concepts are displayed if the entered term matches the beginning of one or more words in the concept’s description, regardless of the order in which those words appear. This allows users to find relevant concepts even if the search term is not at the start of the entire description or the words are entered in a different sequence. For example, entering the term *“dis”* will return concepts like *“Disease”* (where *“Dis”* is the first word) as well as *“Optic disc normal”* (where *“dis”* is the second word), since in both cases the word *“dis”* starts with the entered term.

Upon selecting the focus concept, the corresponding Concept Model Domain is determined via a subsumption testing procedure. This automatically identifies semantically relevant SNOMED CT attributes and their associated value sets. This ensures that the selected attributes and their values comply with the rules defined in the Concept Model. Following this, the user interface is generated dynamically, as shown in Fig. 8. The user then selects the attributes that are relevant for the intended use case. In the example of the focus concept *Disease*, attributes such as *Associated morphology* and *Due to* may be appropriate. For each selected attribute, the user specifies a value set, which can consist of either a single attribute value or multiple values. In the case of multiple values, a logical operator must be defined to specify the relationship between them. When multiple logical operators are used simultaneously, the rules of the ECL grammar require the explicit use of parentheses to ensure syntactic clarity and unambiguous interpretation. To support the user in this process, an informational message is displayed. This message contains a button that opens a modal interface, enabling the user to insert the necessary parentheses. The system automatically validates the placement of the parentheses, and once these are confirmed to be correct, the user can save the updated formulation. All selected values must comply with the defined value range. After successful validation, the corresponding ECL query is generated according to the rules of the Expression Constraint Language. The resulting query can then be either copied to the clipboard or downloaded as a file.

As shown in Fig. 8, an additional panel is displayed alongside the attribute selection interface. This panel presents the definition of the selected attribute as well as its value set. Explicitly providing this attribute information helps users better understand SNOMED CT attributes and facilitates navigation within the concept model.

#### Dashboard

In the upper right corner of the user interface (see Fig. 8), a color-highlighted dashboard labeled *Live* provides real-time feedback on the results of the formulated ECL query. It displays the number of SNOMED CT concepts that have been retrieved based on the current ECL query and the selected terminology version. For example, Fig. 8 shows that the exemplary ECL query – already introduced in the introduction – returned 283 concepts in the chosen SNOMED CT edition and version (International Edition, 2025-01-01). This dashboard offers an immediate indication of whether the constructed query is semantically meaningful and returns relevant results – a crucial aspect when working with ECL query, as an empty result set typically points to a conceptual error in the query. The calculation of the number of matching concepts is performed dynamically in the background and may vary in duration depending on the complexity of the query. Broad or generic queries often result in large concept sets, which can increase response time accordingly. An icon on the right edge of the dashboard allows users to open a modal window that displays all retrieved concepts in detail (see Fig. 8). This detailed view supports verification of the results and facilitates the iterative refinement of the ECL query.

#### SNOMED CT Concept Viewer

**Fig. 9:**
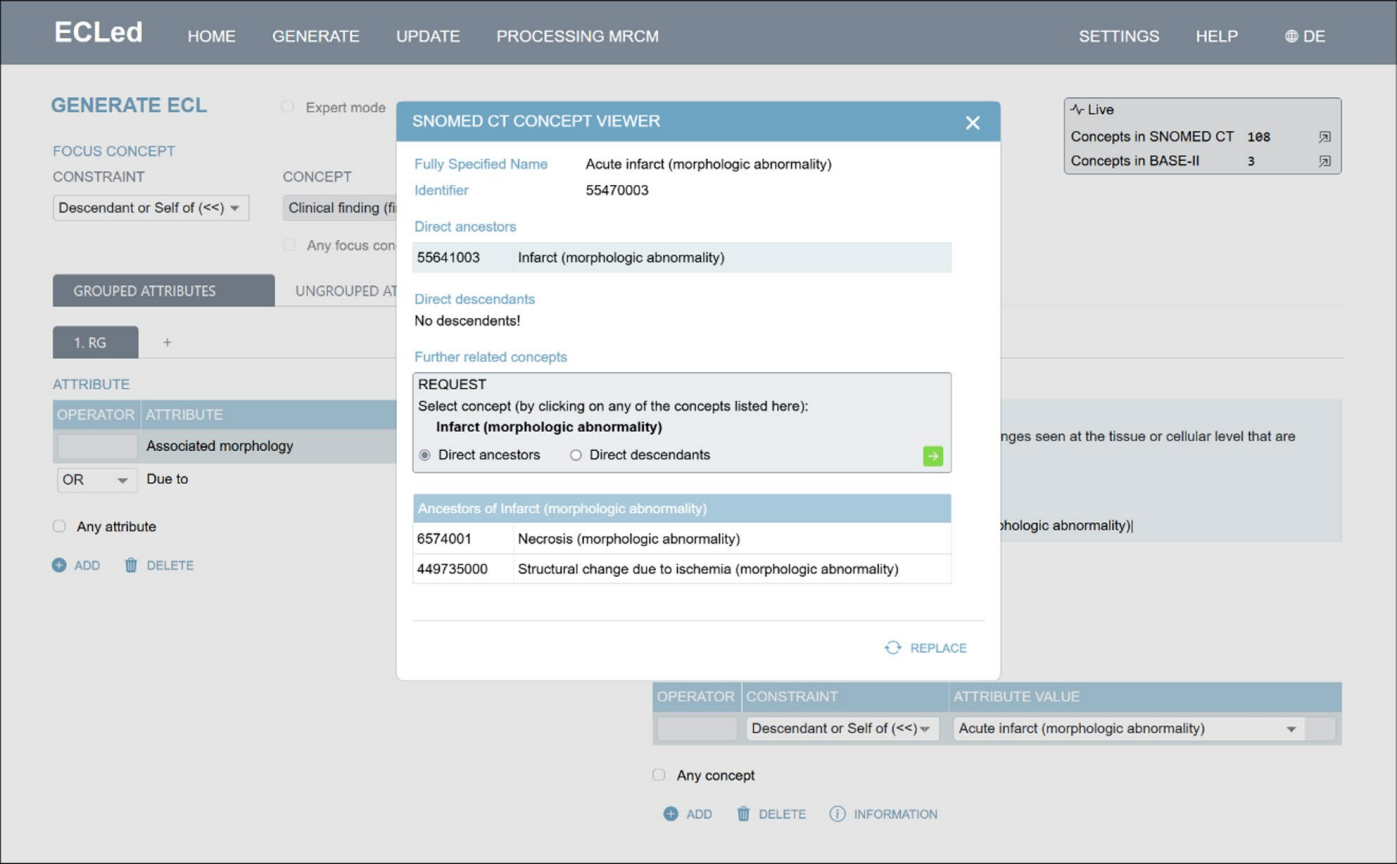
*ECLed*’s SNOMED CT Concept Viewer showing selected concept with parent and child concepts in a modal window

Selecting an appropriate concept is not always straightforward, especially in the context of complex medical scenarios or ambiguous search terms. To support this process, *ECLed* provides a feature for displaying semantically related concepts. This functionality is available in the *Attribute Value* section for a selected attribute via the *Information* button (see Fig. 8, bottom right). Clicking the button opens a modal window (see Fig. 9) that displays the originally selected concept along with its parent and child concepts. Users can further explore these hierarchies by iteratively expanding additional ancestors or descendants of the displayed concepts. If a more suitable concept is identified during this exploration, it can easily be selected as a replacement using the *Replace* button. This feature not only simplifies the selection process but also enhances its quality by enabling users to directly compare and evaluate related concepts in their semantic context.

#### ECL query updating

In addition to creating new ECL query, *ECLed* also enables users to edit existing ones. A previously defined query can be entered into the input field, where it is automatically analysed and loaded into the application. The individual components can then be modified or extended as needed (see section *Create an ECL query*).

### Usability survey

To evaluate the usability of *ECLed*, a custom-designed questionnaire comprising 16 items was developed. The instrument aimed to capture both participants’ overall impressions of the application and their assessment of key functionalities. In addition to two open-ended items for qualitative feedback, 14 questions were answered using a five-point Likert scale (1 = very poor to 5 = very good). Participants also provided a self-assessment of their familiarity with SNOMED CT and the Expression Constraint Language. All participants were aware of available ECL tooling: they were familiar with the *ECL Builder* within the *SNOMED CT Browser* [7], and five participants also had experience with the *Shrimp Browser* [11].

The survey was conducted with eight participants who had basic knowledge of SNOMED CT ECL but no prior experience with *ECLed*. Each participant either explored the tool locally or watched a short demonstration video before completing the questionnaire anonymously. A summary of the quantitative responses is shown in Additional file 1. The evaluation results indicate a high level of user satisfaction with *ECLed*’s usability and core features. Participants consistently rated the interface as well-structured and easy to navigate. Notably, most users were able to construct valid ECL queries without in-depth knowledge of the syntax, suggesting that the tool provides effective guidance and lowers the barrier to entry. Features such as semantic validation of concept combinations, reliable name- and identifier-based concept search, and the hierarchical visualization of related concepts within the Concept Viewer were well received. The ability to save, reload, and export queries worked reliably, and the real-time dashboard displaying the number of matching concepts was perceived as particularly helpful. The interface scaled well for multi-attribute queries, and no significant usability issues were observed in this context. The separation of grouped and ungrouped attributes can present a potential usability issue, as it requires users to understand which attributes are grouped and which are ungrouped, potentially complicating interaction. Overall, participants expressed a willingness to adopt *ECLed* in practical settings, based on the functionalities currently implemented in the tool.

### Validation using real-world ECL queries

#### Data basis

To validate the functionality and correctness of *ECLed*, a total of 31 realistic ECL queries were used (see Additional file 2). Of these, 28 syntactically and semantically correct queries are based on the official *Expression Constraint Language – Specification and Guide* [4] by SNOMED International. These queries include all currently implemented components of the ECL syntax. and serve as the basis for evaluating whether *ECLed* can generate complete and correct ECL queries. In addition, three faulty ECL queries were deliberately constructed to assess *ECLed*’s error detection and prevention mechanisms.

#### Initial validation of data basis

To ensure the validity of the concepts referenced in the queries, all 28 correct ECL queries were checked against the SNOMED CT International Edition, 2025-01-01. A terminology server and the FHIR *$expand* operation were used for this purpose. This combination enables both the resolution and validation of referenced concepts. One of the queries included a deprecated concept (*445238008 |Malignant carcinoid tumor|*), which was replaced with the current equivalent *1288045008 |Well-differentiated neuroendocrine tumor|* based on the Historical Association Reference Sets [4, 25] and the *SNOMED CT Browser* [7].

#### Validation of ECL query creation

The validation showed that all 28 correct ECL queries could be fully reconstructed within *ECLed*. Focus concepts, attributes, attribute values, and constraint operators were consistently available. The generated ECL queries were then automatically compared with the original queries. The results confirmed that all generated queries were syntactically identical to their originals, thereby verifying compliance with both semantic and syntactic rules. In contrast, none of the three intentionally erroneous ECL queries could be recreated in *ECLed*. The invalid elements were not available to the user within the editor, making it impossible to construct the faulty queries.

#### Validation of ECL query updating

In addition, *ECLed*’s second core functionality – updating existing ECL queries – was validated. For this purpose, all 28 correct queries were loaded into the system, automatically parsed, and transferred into the user interface. All elements were correctly recognized, accurately displayed, and could subsequently be modified without restriction.

### Real-world validation using SNOMED CT-coded FHIR data

To evaluate its real-world applicability, the tool *ECLed* was applied to clinical patient data encoded in the FHIR format and containing diagnoses coded with SNOMED CT. The aim of this validation was to assess the extent to which relevant concepts can be reliably identified in real-world datasets using ECL queries generated by *ECLed* (see Additional file 3). For querying, the integrated FHIR Search functionality of *ECLed* was used, which enables automated semantic queries against FHIR data. The dataset originates from the German Berlin Aging Study II (BASE-II), a multidisciplinary longitudinal study focused on health-related aging processes [26]. The dataset comprises 1,295 pseudonymized patients and includes the FHIR resources *Condition* and *Provenance*, both of which are central to this work. The *Condition* resources contain a total of 1,674 unique SNOMED CT concepts, among other coding systems. The *Provenance* resources document the origin and further processing of clinical information automatically extracted from medical report and mapped to various terminologies, including SNOMED CT. For the validation (see Additional file 3), a representative use case was defined: “Identifying findings indicative of infarct-like morphological changes”. To this end, the following ECL query was generated using *ECLed*:

*<< 64572001 |Disease (disorder)|*:

*{ 116676008 |Associated morphology (attribute)| =*

*<< 55641003 |Infarct (morphologic abnormality)|*.

This query was intentionally kept simple, as previous studies had already demonstrated *ECLed*’s ability to generate more complex queries correctly.

Subsequently, it was analyzed which specific SNOMED CT concepts in the BASE-II dataset matched this query. A total of six relevant concepts were identified (see Appendix 3). To complement this, the *Provenance* resources were evaluated to determine which entries explicitly contained the German term *“Infarkt”* (English: infarct) within their free-text annotations. This analysis served to further validate the semantic plausibility of the identified concepts. As a result, five concepts were identified that matched both the ECL query and the linguistic evidence in the annotations (see Appendix 3). This indicates a high degree of consistency between the concepts retrieved by ECLed and those confirmed through Provenance analysis, with five out of six relevant concepts detected by both approaches. The only exception was the SNOMED CT concept *22,298,006 |Myocardial infarction (disorder)|*, which was not supported by the Provenance data, as the corresponding annotation merely referred to *“angina pectoris”* without explicitly mentioning *“Infarkt”*.

## Discussion

### Principle findings

The primary objective of this work was to develop *ECLed*, a specialized tool designed to lower entry barriers to the SNOMED CT Expression Constraint Language (ECL) and facilitate semantic querying of clinical data for a broad range of users. As highlighted in the introduction, the formal complexity of ECL presents significant challenges, particularly for users with limited experience in medical terminologies or query languages. *ECLed* addresses these challenges through a web-based platform featuring intuitive interface, visualization of semantic structures, and automatic generation of syntactically valid ECL queries. By adhering to the official ECL grammar and integrating the SNOMED CT Concept Model, the tool ensures both syntactic correctness and semantic validity. The open-source availability of *ECLed* on Gitlab [27] encourages community-driven development. A first real-world integration has been achieved within the *DaWiMed* research platform developed by ID GmbH & Co. KGaA [9], where *ECLed* enables domain experts to define precise semantic query constraints. In this integration, *ECLed* can be opened in a separate tab within *DaWiMed*, allowing users to create ECL queries and easily transfer them back into the platform using the copy-to-clipboard function. Its simplicity and lightweight design facilitate straightforward integration into other systems and workflows, highlighting its applicability not only in clinical research but also in broader data exploration contexts. The implementation leverages modern web technologies alongside an embedded FHIR terminology server (e.g., *Ontoserver* [22] or *SNOWSTORM* [23]), allowing seamless integration with standardized FHIR services. Although *ECLed* currently operates with the SNOMED CT International Edition (2025-01-01), it supports switching to other editions or versions, provided the corresponding MRCM is regenerated in JSON format. This process is facilitated by the freely available web application *WASP* [8, 17, 28], developed in a previous project.

Compared to tools such as the *ECL Builder* integrated in the *SNOMED CT Browser* [7] or *Shrimp* of CSIRO [11], *ECLed* addresses specific limitations by fully implementing the rules of the Concept Model (similar to the *ECL Builder*) and improving usability for non-technical users. Unlike *Shrimp*, which offers a broader set of features but does not enforce Concept Model conformance, *ECLed* ensures compliance with the model. Plans are in place to extend its functionality to cover additional components supported in *Shrimp* (see Section Implementation Strategy). Feedback from a usability survey indicated high satisfaction with the interface and functionality, emphasizing ease of use in constructing syntactically and semantically correct ECL queries and visualizing hierarchical relationships within the Concept Viewer. The survey also highlighted a potential usability issue: the separation of grouped and ungrouped attributes may require users to understand which attributes fall into each category, potentially complicating interaction. A user-friendly implementation of this feature should be considered in future versions. Technical validation demonstrated that *ECLed* reliably handles complex ECL queries. Validation tests using 38 official examples from the *Expression Constraint Language – Specification and Guide* [4] confirmed that all implemented components were correctly represented and syntactically valid. The tool prevented invalid queries and maintained stable update functionality for existing queries. *ECLed* was further tested with clinical data in FHIR format from the Berlin Aging Study II [26], successfully identifying relevant concepts and executing ECL queries as intended, confirming functional integration with a native FHIR server. Scalability has not yet been systematically evaluated, and large-scale testing is needed to assess performance in production environments. In summary, most requirements (see Table 1) were met. Usability (N1) was confirmed through user feedback, and functionality (N2) was validated empirically and technically. Maintainability (N4) was ensured by separating frontend and backend components, and portability (N5) was confirmed across different systems and browsers. While performance (N3) has not been formally benchmarked, current experiments do not indicate major issues. Response times may vary depending on the terminology server and query specificity, with broader value ranges (e.g., Due To) potentially increasing processing times. All key functional requirements (F1–F5) were successfully implemented and validated, particularly with the terminology server and FHIR integration.

### Implementation strategy and prioritization

At the start of this work, the ECL grammar was reviewed with domain experts, and its components were prioritized (see Fig. 3). Out of a total of 38 identified components, 23 were classified as essential, as they constitute the foundational elements required for basic functionality. All of these were successfully implemented, as confirmed in the subsequent validation. In addition, three further components from the *Desirable* category were fully implemented, including the use of parentheses when combining multiple logical operators and the reverse function. One additional element was partially realized. The implementation of cardinalities has been initiated but is not yet complete, as it is implemented in *Shrimp*. At present, only the *[0..0]* cardinality is supported, as it represents negation. This a commonly used approach, alongside the *“!=”* comparison operator, for expressing exclusions. Components classified as *Currently Not Relevant* were not implemented in this work due to their higher complexity. This category includes features such as multiple focus concepts, which are supported by both *ECL Builder* [7] and *Shrimp* [11], and nested expression constraints, which are only available in *Shrimp* [11]. Implementing these features at an early stage would have required substantial additional effort and increased the risk of errors. Instead, the focus was placed on establishing a stable foundation of essential and desirable components, ensuring that the core functionality and user interface could be validated effectively. This approach also allows for an iterative extension of the system: once the basic operations are stable and user interactions are well understood, more complex features can be integrated in a controlled and user-centered manner. Additionally, prioritizing commonly used functions ensures that development resources are allocated to features that provide the greatest practical benefit. The components *Description*, *Member*, and *Concept Filter* were also classified as *Currently Not Relevant*, as they do not contribute directly to the retrieval of patient data and were therefore considered outside the scope of this work. Some relevant features, such as *History Supplements* or Reference Sets (implemented in *Shrimp* [11]), have not yet been implemented and are marked as *Future Implementation* in Fig. 3. This choice was made to prioritize a simplified representation of the most essential elements, focusing on supporting straightforward search and identification of concepts in medical data that match an ECL query.

### Future work

A key aspect of future research is the integration of *History Supplements* [4], which enable tracking changes and managing versioning within SNOMED CT. This is an increasingly important task given the continual evolution of medical terminologies. Incorporating these elements into future versions of *ECLed* would enhance data consistency and quality assurance in SNOMED CT-encoded datasets. Alternatively, History-ECL queries could be used to capture historical variants of long-standing concepts without replacing deprecated concepts, although this approach may be problematic for some domain experts. Implementing history-related reference sets, such as *900000000000527005 |SAME AS association reference set|*, along with the corresponding member filters, would facilitate version tracking within SNOMED CT. Furthermore, mechanisms to query historical data based on predefined profiles, such as *HISTORY-MIN*, should be provided. Realizing these features will require careful design and a user-friendly interface to ensure that users can effectively explore and manage the evolution of concepts over time. Another potential extension would be the support for Reference Sets in ECL queries, allowing for more flexible handling of SNOMED CT concepts. Reference Sets enable grouping or selection of specific subsets of concepts, which is particularly useful for local adaptations, quality measures, or historical tracking. Integrating ReferenceSets into ECL queries would allow users to target these subsets directly, improving query efficiency and ensuring consistency in data retrieval. Technically, this requires incorporating member filters and mechanisms to combine ReferenceSets with logical operators and attribute constraints. A user-friendly interface would also be necessary to allow clinicians and analysts to select and manage ReferenceSets effectively within queries.

Additionally, future work could focus on providing a natural language representation of ECL queries. This feature would enable users to intuitively verify the intended meaning of a query in a human-readable format. This could reduce the risk of semantic errors and increase trust in the query results, especially for users without a technical background. For example, a user creates the following ECL query:

*<< 64572001 |Disease (disorder)|*:

*{ 363698007 |Finding site (attribute)| =*

*<< 119199005 |Lung part (body structure)| }*.

This query could be rendered in natural language as:

*A disease that affects a part of the lung.*

If the user’s actual intention was to express a condition affecting the entire lung structure or a higher-level anatomical region, this discrepancy would become apparent through the natural language output. In such a case, the user might reconsider using *39607008 |Lung structure (body structure)|* as the attribute value instead. Thus, natural language rendering not only improves comprehensibility but also helps enhance the accuracy and quality of ECL query formulation.

## Conclusion

*ECLed* lowers the barrier to working with SNOMED CT ECL by combining a user-friendly interface with integrated semantic validation and a modular, standards-based architecture. Developed based on a comprehensive requirements analysis, the tool enables the reliable construction and editing of ECL queries, even for non-technical users. Its integration into real-world platforms like *DaWiMed* highlights its practical relevance. *ECLed* improves accessibility to semantic querying in clinical research, providing a solid foundation for further technical advancements while enhancing the error-resistant construction and semantic accuracy of SNOMED CT-based clinical data queries.

## Supplementary Information

Below is the link to the electronic supplementary material.

Supplementary Material 1: Usability Survey – Results

Supplementary Material 2: Validation using real-world ECL queries

Supplementary Material 3: Real-world validation using SNOMED CT-coded FHIR data

## Data Availability

Project name: ECLed, Project home page: [https://gitlab.com/tessa00/ecl-editor](https:/gitlab.com/tessa00/ecl-editor) (Interested parties may contact the corresponding author for further information), Operating system: Platform independent, Programming languages: Java, Spring Boot, Angular, Other requirements: Java 17, SpringBoot 3.3.2, Agular 18.2.9, Maven, License: Apache License 2.0, Any restrictions to use by non-academics: None apply, Additional notes: The FHIR data from the BASE-II study are not publicly available due to privacy regulations.

## **Abbreviations**

ANTLR: A Nother Tool for Language Recognition
CSIRO: Commonwealth Scientific and Industrial Research Organisation
ECL: Expression Constraint Language
FHIR: Fast Healthcare Interoperability Resources
HL7: Health Level Seven
ICD-10: International Statistical Classification of Diseases and Related Health Problems, 10th Revision
JSON: JavaScript Object Notation
MRCM: Machine Readable Concept Model
OPS: Operationen- und Prozedurenschlüssel
PCE: Postcoordinated Expression
REST: Representational State Transfer
SNOMED CT: SNOMED Clinical Terms
WASP: Web Application Security Project
HTTP: Hypertext Transfer Protocol
