# Supplementary material for: ECLed– a tool supporting the effective use of the SNOMED CT Expression Constraint Language
Source: J Biomed Semantics. 2026 Jan 6;17:1. doi: 10.1186/s13326-025-00344-3 (PMC12777381; doi:10.1186/s13326-025-00344-3)
Supplement: Supplementary file 1 — Supplementary Material 1: Usability Survey – Results [file 13326_2025_344_MOESM1_ESM.docx]

**Usability Survey – Results**

**Two more open-ended questions:**

- Do you have any suggestions on how users could be further supported when creating an ECL query?
- Further feedback
