# Supplementary material for: ECLed– a tool supporting the effective use of the SNOMED CT Expression Constraint Language
Source: J Biomed Semantics. 2026 Jan 6;17:1. doi: 10.1186/s13326-025-00344-3 (PMC12777381; doi:10.1186/s13326-025-00344-3)
Supplement: Supplementary file 2 — Supplementary Material 2: Validation using real-world ECL queries [file 13326_2025_344_MOESM2_ESM.docx]

**Validation Using Real-World ECL Queries**

|  | **ECL query** | **Major characteristics** |
| --- | --- | --- |
| **CORRECT ECL** | * | - Only focus concept (Any)  [Any] |
|  | 404684003 \|Clinical finding\| | - Only focus concept  [Self] |
|  | << 73211009 \|Diabetes mellitus\| | - Focus concept + constraint operator  [Descendant or Self of] |
|  | < 404684003 \|Clinical finding\| | - Focus concept + constraint operator  [Descendant of] |
|  | !>> 404684003 \|Clinical finding\| | - Focus concept + constraint operator  [Child or Self of] |
|  | !> 404684003 \|Clinical finding\| | - Focus concept + constraint operator  [Child of] |
|  | >> 40541001 \|Acute pulmonary edema\| | - Focus concept + constraint operator  [Ancestor or Self of] |
|  | > 40541001 \|Acute pulmonary edema\| | - Focus concept + constraint operator  [Ancestor of] |
|  | >>! 40541001 \|Acute pulmonary edema\| | - Focus concept + constraint operator  [Parent or Self of] |
|  | >! 40541001 \|Acute pulmonary edema\| | - Focus concept + constraint operator  [Parent of] |
|  | < 19829001 \|Disorder of lung\|:  {116676008 \|Associated morphology\| = 79654002 \|Edema\|} | - 1 RoleGroup with 1 attribute relation  [Attribute group, Comparison operator] |
|  | < 19829001 \|Disorder of lung\|:  {116676008 \|Associated morphology\| = << 79654002 \|Edema\|} | - Using constraint operator before attribute   value  [Descendant and Self of bei Attributwert] |
|  | < 404684003 \|Clinical finding\|: {* = 79654002 \|Edema\|} | - Using Any (*) as attribute  [Any attribute] |
|  | < 404684003 \|Clinical finding\|: {116676008 \|Associated morphology\| = *} | - Using Any (*) as attribute value  [Any value] |
|  | < 404684003 \|Clinical finding\| :  116676008 \|Associated morphology\| != << 26036001 \|Obstruction\| | - Using Not-equals as comparison operator  [Comparison operator – Not-equals] |
|  | < 404684003 \|Clinical finding\| :  [0..0] 116676008 \|Associated morphology\| = << 26036001 \|Obstruction\| | - Negation  [Cardinality] |
|  | < 404684003 \|Clinical finding\|:  {363698007 \|Finding site\| = << 39057004 \|Pulmonary valve structure\| AND   116676008 \|Associated morphology\| = << 415582006 \|Stenosis\|} | - 1 RoleGroup with 2 attribute relation - Logical operator between relations: AND  [RoleGroup Conjunction] |
|  | < 404684003 \|Clinical finding\|:  {116676008 \|Associated morphology\| = << 55641003 \|Infarct\| OR  42752001 \|Due to\| = << 22298006 \|Myocardial infarction\| | - 1 RoleGroup with 2 attribute relation - Logical operator between relations: OR  [RoleGroup Disjunction] |
|  | < 404684003 \|Clinical finding\|:  {363698007 \|Finding site\| = << 39057004 \|Pulmonary valve structure\| AND  116676008 \|Associated morphology\| = << 415582006 \|Stenosis\|} AND  {363698007 \|Finding site\| = << 53085002 \|Right ventricular structure\| AND  116676008 \|Associated morphology\| = << 56246009 \|Hypertrophy\|} | - 2 RoleGroups with 2 attribute relation  - Logical operator between RoleGroups: AND  [Attribute relation Conjunction] |
|  | < 404684003 \|Clinical finding\|:  {363698007 \|Finding site\| = << 39057004 \|Pulmonary valve structure\| OR  116676008 \|Associated morphology\| = << 415582006 \|Stenosis\|} OR  {363698007 \|Finding site\| = << 53085002 \|Right ventricular structure\| OR  116676008 \|Associated morphology\| = << 56246009 \|Hype} | - 2 RoleGroups with 2 attribute relation  - Logical operator between RoleGroups: OR  [Attribute relation Disjunction] |
|  | < 373873005 \|Pharmaceutical / biologic product\|:  411116001 \|Has manufactured dose form\|= << 385268001 \|Oral dose form\| | - 1 ungrouped attribute relation  [Choose attribute – ungrouped] |
|  | < 763158003 \|Medicinal product\|:  411116001 \|Has manufactured dose form\| = << 385268001 \|Oral dose form\| AND  {127489000 \|Has active ingredient\| = << 372687004 \|Amoxicillin\| AND  1142135004 \|Has presentation strength numerator value\| >= #250 AND  732945000 \|Has presentation strength numerator unit\| = 258684004 \|milligram\|} | - 1 ungrouped attribute relation  - 1 RoleGroup with 2 attribute relation  - Attribute relation with concrete value  [Logical operator, Concrete value] |
|  | < 763158003 \|Medicinal product\|:  411116001 \|Has manufactured dose form\|= << 385268001 \|Oral dose form\| AND  {127489000 \|Has active ingredient\| = << 372687004 \|Amoxicillin\| AND  1142135004 \|Has presentation strength numerator value\| >= #250 AND  1142135004 \|Has presentation strength numerator value\| <= #800 | - 1 ungrouped attribute relation  - 1 RoleGroup with 2 attribute relation  - Attribute relation with concrete value (min   and max value)  [Concrete value – min/max] |
|  | < 91723000 \|Anatomical structure\|:  R 363698007 \|Finding site\| = < 125605004 \|Fracture of bone\| | - Reverse attribute  [Reverse] |
|  | < 404684003 \|Clinical finding\|:  {(363698007 \|Finding site\| = << 39057004 \|Pulmonary valve structure\| AND  116676008 \|Associated morphology\| = << 415582006 \|Stenosis\|) OR  42752001 \|Due to\| = << 1288045008\|Well-differentiated neuroendocrine tumor\|} | - Multiple logical operators between  relations: AND / OR (brackets)  [Brackets – Conjunction/Disjunction] |
|  | < 404684003 \|Clinical finding\|:  {363698007 \|Finding site\| = << 39057004 \|Pulmonary valve structure\| AND  (116676008 \|Associated morphology\| = << 415582006 \|Stenosis\| OR  42752001 \|Due to\| = << 1288045008\|Well-differentiated neuroendocrine tumor\|)} | - Multiple logical operators between  relations: AND / OR (brackets)  [Brackets – Conjunction/Disjunction] |
|  | < 404684003 \|Clinical finding\|:  {116676008 \|Associated morphology\| = ((<< 56208002 \|Ulcer\| AND << 50960005 \|Hemorrhage\|) MINUS << 26036001 \|Obstruction\|)} | - Multiple logical operators between attribute   values: AND / MINUS (brackets)  [Brackets – Conjunction/Exclusion] |
|  | < 404684003 \|Clinical finding\|:  {116676008 \|Associated morphology\| = ((<< 56208002 \|Ulcer\| AND << 50960005 \|Hemorrhage\|) MINUS <<  26036001 \|Obstruction\|)} | - Multiple logical operators between attribute   values: AND / OR (brackets)  [Attribute value range – Conjunction/Exclusion] |
| **INCORRECT ECL** | < 404684003 \|Clinical finding\|:  {363704007 \|Procedure site\| = << 39057004 \|Pulmonary valve structure\|} | - Use of a invalid attribute (*Procedure site*) in   the domain *Clinical finding* |
|  | < 404684003 \|Clinical finding\|:  {363698007 \|Finding site\| = << 39057004 \|Pulmonary valve structure\|} MINUS  363698007 \|Finding site\| = << 53085002 \|Right ventricular structure\|} | - Violation of the rules of logical operators with respect to the RoleGroups – only AND and OR are permitted. |
|  | < 404684003 \|Clinical finding\|:  {363698007 \|Finding site\| = << 90734009 \|Chronic\|} | - Violation of an attribute's range through the   selection of an invalid attribute value (e.g.,   *Chronic*) |

**Note**:

The 18 syntactically and semantically correct ECL queries are taken from the official *Expression Constraint Language – Specification and Guide* [4] by SNOMED International, to demonstrate that *ECLed* can generate correct and precise ECL expressions. The three incorrect ECL queries, on the other hand, were intentionally created by us to illustrate that *ECLed* can detect and preventing errors. The right column of the table shows the characteristics of the individual ECL expressions. In addition, the elements from the requirements analysis (see Figure 3) are listed in blue square brackets. For the sake of clarity, not all elements were listed for each ECL query; instead, care was taken to ensure that all elements are covered overall.
