# Supplementary material for: ECLed– a tool supporting the effective use of the SNOMED CT Expression Constraint Language
Source: J Biomed Semantics. 2026 Jan 6;17:1. doi: 10.1186/s13326-025-00344-3 (PMC12777381; doi:10.1186/s13326-025-00344-3)
Supplement: Supplementary file 3 — Supplementary Material 3: Real-world validation using SNOMED CT-coded FHIR data [file 13326_2025_344_MOESM3_ESM.docx]

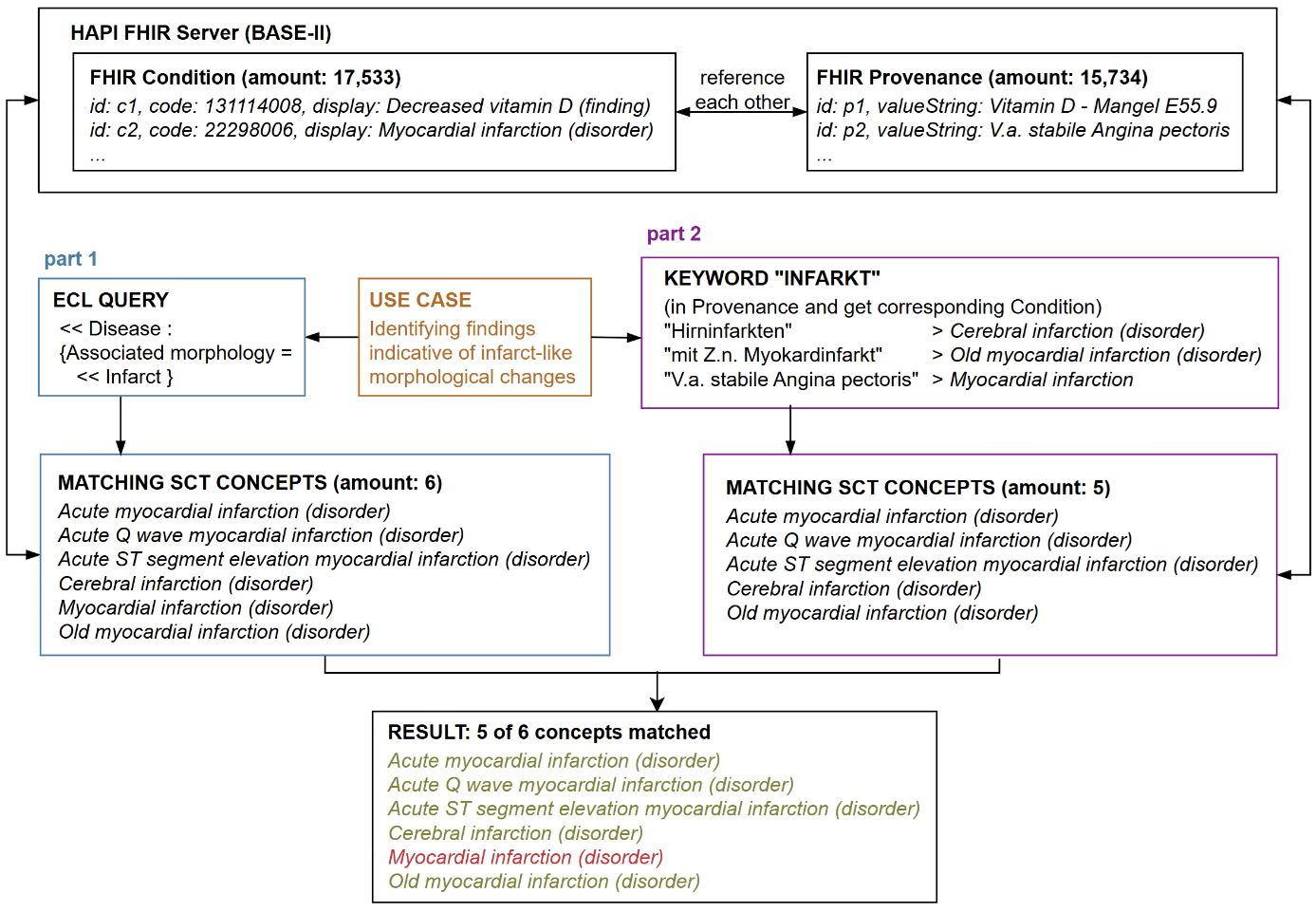


**Real-World Validation Using SNOMED CT-Coded FHIR Data**

**Figure 1.** Comparison of Semantic and Keyword-Based Methods for Identifying Infarct-Associated Clinical Concepts in FHIR Data.

This figure illustrates two different approaches for identifying clinically relevant, infarct-associated conditions within a FHIR dataset (BASE-II) using SNOMED CT:

- **Part 1 – Semantic Query (left):**

A precisely formulated Expression Constraint Language (ECL) query leverages SNOMED CT relationships to extract disorders characterized by infarct-type morphological changes. This query yields five matching concepts.

- **Part 2 – Keyword Search (right):**

The keyword “Infarkt” (German for infarction) is searched within the textual content of *Provenance* entries. This method identifies six concepts in total.

- **Conclusion:**

Both methods identify five overlapping SNOMED CT concepts, including cerebral infarction. While the semantic ECL query ensures high precision, keyword-based extraction from narrative provenance text can uncover additional relevant conditions. Combining both approaches can improve completeness and accuracy in the identification of infarct-related clinical concepts from structured and unstructured healthcare data.
